# When life comes to an end: lessons from microbial aging models

**Published:** 2010-08-16

**Authors:** Heinz D. Osiewacz

**Affiliations:** Johann Wolfgang Goethe University, Faculty for Biosciences & Cluster of Excellence Macromolecular Complexes, Institute of Molecular Biosciences, 60438 Frankfurt, Germany

Aging of biological systems ultimately leads to death of the individual. In humans, organ failure as the result of functional impairments after stroke, cardio-vascular disease, tumor development, neurodegeneration and other diseases are certainly crucial in bringing life to an end. But what happens in individuals with no obvious disease or disorders? In simple microorganisms like the single cellular fungus *Saccharomyces cerevisiae* (yeast) very basic cellular processes, resembling mammalian programmed cell death (PCD) as they are important for development and protection against cancer, lead to death of the individuum [1,2]. In the last several years, PCD was also demonstrated to occur in multicellular fungi [2]. In the filamentous fungus *Podospora anserina*, a well established experimentally tractable short-lived aging model with a small genome, a role of PCD was recently demonstrated to be involved in aging and lifespan control [3-5]. Although differing in complexity, the molecular pathways controlling PCD in the different groups of organisms are conserved [6,7]. In a recent study we addressed the role of PaCYPD (cyclophilin D) a *P. anserina* [8], a mitochondrial matrix protein previously found to increase in abundance in mitochondria during aging  [9]. Cyclophilin D is a peptidyl prolyl-*cis,trans*-isomerase located in the mitochondrial matrix that is known to be involved in mitochondrial permeability pore transition (mPTP) [10], a process leading to opening of a membrane pore and to entrance of low molecular weight solutes into the mitochondrial matrix. The process leads to swelling of mitochondria, rupture of the outer membrane and the release of apoptogens (e.g., cytochrome c) from the intermembrane space and the subsequent induction of PCD. We now show that over-expression of *PaCypD* results in accelerating organismal aging. Chronological young *PaCypD* overexpressors display a ‘premature aging' phenotype with pronounced mitochondrial dysfunction, markers of senescence and apoptosis, and reduced stress tolerance. Treatment with the CYPD inhibitor cyclosporin A leads to correction of mitochondrial function and lifespan to that of the wild-type.

It should be emphasized here that the induction of PCD as a final execution program in the life cycle of the fungus appears to result from complex aging processes. In *P. anserina*, a number of different pathways including those leading to the generation and scavenging of reactive oxygen species [11-14], protein quality control [15], mitochondrial dynamics [16], and others [3,4] are known to be part of the complex network governing aging.

It is now the challenge to identify other components of mitochondrial pore formation, characterize the pore biochemically, and to elucidate the detailed role of PCD in aging also in mammalian systems. Interestingly, an age-related increase of CYPD, as in *P. anserina*, was recently reported in rat gastrocnemius muscle and in brain tissue of mice and humans [17,18]. It is thus likely that the induction of PCD, at least in particular tissues and organs, also in human aging plays a more important role than it is currently anticipated. If so, it will be of special interest to investigate the potential of components like CSA, a cyclic peptide approved for human therapy in transplantation medicine or for treatment of Ullrich congenital muscular dystrophy, in being effective in postponing basic aging processes and using this knowledge to develop efficient strategies to intervene into biological aging.

## References

[1] Madeo F (2004). Curr Opin Microbiol.

[2] Hamann A, Brust D, Osiewacz HD (2008). Trends Microbiol.

[3] Osiewacz HD, Clairmont A, Huth M (1990). Curr Genet.

[4] Scheckhuber CQ (2007). Nat Cell Biol.

[5] Osiewacz HD (2007). Mech Ageing Dev.

[6] Hamann A, Brust D, Osiewacz HD (2007). Mol Microbiol.

[7] Brust D, Hamann A, Osiewacz HD (2010). Curr Genet.

[8] Brust D (2010). Aging Cell.

[9] Groebe K (2007). Exp Gerontol.

[10] Azzolin L (2010). FEBS Lett.

[11] Gredilla R, Grief J, Osiewacz HD (2006). Exp Gerontol.

[12] Kunstmann B, Osiewacz HD (2008). Aging Cell.

[13] Kunstmann B, Osiewacz HD (2009). Aging.

[14] Knab B, Osiewacz HD (2010). Cell Cycle.

[15] Luce K, Osiewacz HD (2009). Nat Cell Biol.

[16] Scheckhuber CQ, Osiewacz HD (2008). Mol Genet Genomics.

[17] Du H (2008). Nat Med.

[18] Marzetti E (2008). Mech Ageing Dev.

